# Association between intrinsic capacity and dementia risk in older Mexicans

**DOI:** 10.1002/alz.71578

**Published:** 2026-06-17

**Authors:** Miriam Teresa López‐Teros, Sara Gabriela Yeverino‐Castro, Fabiola Yocupicio‐ Medrano, Alberto José Mimenza‐Alvarado, Luis Miguel Gutiérrez‐Robledo, Omar Yaxmehen Bello‐Chavolla, Sara Gloria Aguilar‐Navarro

**Affiliations:** ^1^ Department of Nutrition Instituto Nacional de Ciencias Médicas y Nutrición Salvador Zubirán Mexico City Mexico; ^2^ Geriatrics Service Universidad Autónoma de Nuevo León Dr. José E. González University Hospital Monterrey Nuevo León México; ^3^ Department of Geriatric Medicine Instituto Nacional de Ciencias Médicas y Nutrición Salvador Zubirán Tlalpan, Mexico City Mexico; ^4^ Department of Clinical Epidemiology Instituto Nacional de Geriatría Mexico City Mexico

**Keywords:** cognitive impairment, dementia, healthy aging, Intrinsic capacity, older adults

## Abstract

**INTRODUCTION:**

Healthy aging reflects the maintenance of intrinsic capacity (IC) across five physical and mental domains. Although IC decline has been linked to frailty and mortality, evidence regarding its predictive value for dementia remains limited, particularly in low‐ and middle‐income countries.

**METHODS:**

Data from 7263 adults aged ≥60 years without baseline dementia from the Mexican Health and Aging Study (2012–2015) were analyzed. IC was assessed across vitality, locomotor, sensory, cognitive, and psychological domains. Dementia was defined as impairment in two or more cognitive domains plus one or more limitation in instrumental activities of daily living.

**RESULTS:**

The mean age of the study population was 68.92 ± 6.8 years and 55.4% were women. Dementia incidence over 3 years was 4.15%. Higher baseline IC scores were associated with lower odds of incident dementia (adjusted odds ratio [OR] = 0.62; 95% confidence interval [CI]: 0.41–0.94).

**DISCUSSION:**

Our findings support the role of IC as an early, multidimensional marker of cognitive vulnerability.

## Highlights

- Intrinsic capacity (IC) was associated with 3‐year dementia risk in older Mexican adults.
- Higher baseline IC corresponded to lower odds of incident dementia.
- IC captured multidimensional resilience beyond cognitive performance alone.
- IC was associated with early markers of cognitive vulnerability.
- Differences in IC were associated with dementia risk stratification in resource‐limited settings.

## 1 BACKGROUND

Dementia is a clinical syndrome characterized not only by memory impairment but also by deficits in attention, language, and other cognitive domains, ultimately affecting daily functioning.^1^ It poses an escalating global public health challenge, with an estimated 57 million people living with dementia worldwide in 2021, a number projected to rise substantially over the next few decades, particularly in low‐ and middle‐income countries.^2^ Worldwide, dementia affects roughly 5%–7% of individuals 60 years and older, with prevalence rising significantly—to approximately 21% among those between 80 and 89 years.^3^ In Mexico, national data indicate a dementia prevalence of around 6% among adults 60 years and older, with an incidence of approximately 27 per 1000 person‐years.^4^, ^5^ This growing burden underlines the importance of identifying potential longitudinal predictors such as intrinsic capacity (IC) for the design of preventive strategies, as declines or deficits in IC have been associated with an increased risk of dementia.^6^ Broadly defined, IC reflects the aggregate of an individual's physical and mental abilities that sustain functional capacity and healthy aging.^7^

IC is conceptualized into five domains—cognition, vitality, locomotion, psychological well‐being, and sensory function—which can be quantitatively assessed using the World Health Organization‘s (WHO) Integrated Care for Older People (ICOPE) guidelines as a holistic measure of older adults’ health.^8^

IC differs from concepts such as frailty, which primarily identify deficits and decline; instead, IC emphasizes capacities that can be maintained or strengthened, highlighting a strengths‐based perspective on aging.^9^ As a composite construct, IC integrates multiple interrelated domains that jointly reflect overall functional reserve, offering a multidimensional summary of aging‐related vulnerability beyond domain‐specific predictors.^10^ By capturing the dynamic interplay between an individual's capacities and their surrounding environment, including social contexts, attitudes, values, and policies, IC provides a framework to understand resilience and vulnerability to adverse outcomes such as cognitive decline and dementia.^11^

One of the critical challenges in aging research is the need for reliable strategies to monitor individual aging trajectories and predict vulnerability to age‐related health problems.^12^ As individuals age, however, physiological changes become more prevalent, increasing the risk of declines in IC and the development of multimorbidity.^8^ There has been increasing interest in this concept, as accumulating evidence highlights its role as a predictor of adverse outcomes such as disability, falls, hospitalization, mortality, frailty, and other health conditions including oral‐related outcomes.^13^, ^14^, ^15^, ^16^ Evidence linking IC deficits to dementia, however, remains scarce. To date, only a prospective analysis from the United Kingdom Biobank has demonstrated that older adults with greater IC deficits exhibited up to a two‐fold higher risk of developing dementia compared with those without IC impairments.^6^

Notwithstanding, most of the available evidence on IC originates from high‐income settings, predominantly in Europe and Asia, whereas studies from Latin America and other low‐ and middle‐income countries remain scarce. Moreover, the few studies conducted in these regions have primarily focused on cross‐sectional or prevalence analyses, with a notable lack of longitudinal research exploring trajectories of IC and its long‐term implication.^17^, ^18^ Understanding predictors of dementia and other health outcomes, including IC, is critical for designing effective prevention strategies and informing health policy in the Mexican context, particularly given the rapid demographic transition marked by population aging, high dementia prevalence, and limited health resources.^19^ The Mexican Health and Aging Study (MHAS) enables the examination of these questions using nationally representative, longitudinal data with validated cognitive measures.^20^

Despite global adoption of IC, its predictive validity for dementia has not been sufficiently tested in Mexican older adults using large population‐based data. Therefore, the aim of this study was to evaluate the association between baseline IC and risk of dementia over a 3‐year follow‐up among older Mexican adults using the MHAS 2012–2015 cohort.

## 2 METHODS

### 2.1 Study participants and design

The MHAS is a nationally representative cohort of adults 50 years and older in Mexico. The study began in 2001 and has since included five follow‐up waves (2003, 2012, 2015, 2018, and 2021). For the present analysis, we used cross‐sectional data from the 2012 wave, along with a subsample from 2015.

Detailed information on the study design and ethical approval is available elsewhere.^21^, and its aims and methodological framework have been published previously.

### 2.2 Sample selection at baseline and follow‐up

**RESEARCH IN CONTEXT**

1. **Systematic review**: The literature was reviewed through PubMed and Scopus using terms related to “intrinsic capacity,” “dementia,” “cognitive decline,” and “older adults.” Previous studies have demonstrated that declines in intrinsic capacity (IC) are associated with frailty, disability, and mortality. However, only a few longitudinal studies have examined its predictive value for dementia, and none have focused on the Mexican population or other low‐ and middle‐income countries.
2. **Interpretation**: Using nationally representative data from Mexican older adults, this study found that higher baseline IC was associated with lower odds of incident dementia over 3 years, independent of sociodemographic and health covariates. These findings are consistent with IC as an early, multidimensional marker of cognitive vulnerability.
3. **Future directions**: Future research should examine the mechanisms linking IC decline and dementia, assess whether interventions targeting IC influence dementia risk, and explore the integration of IC into public health strategies for risk stratification and the promotion of healthy aging.

**FIGURE 1:**
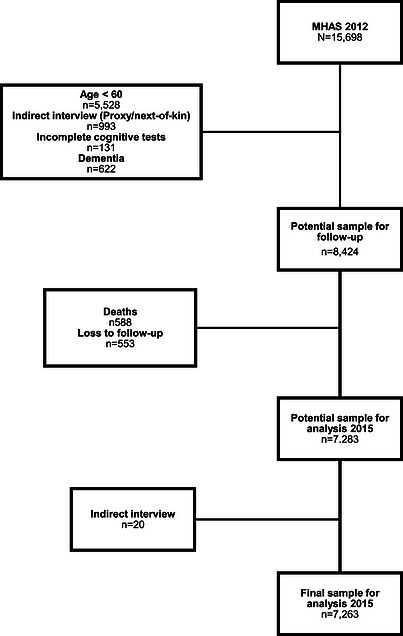
Flowchart of participant selection from the MHAS, 2012–2015. MHAS, Mexican Health and Aging Study.

Figure 1 presents the flow chart of the study population derived from the MHAS. From the original 2012 survey sample of 15,698 adults, 5528 participants younger than 60 years were excluded, as the present study focused on older adults, consistent with the WHO of older age in low‐ and middle‐income countries and the target population of the ICOPE framework.^8^ Additionally, 993 proxy or next‐of‐kin interviews, 131 participants with incomplete cognitive tests, and 622 participants with dementia were excluded, leaving 8424 eligible participants for follow‐up. Between 2012 and 2015, 588 participants died and 553 were lost to follow‐up or refused participation, resulting in 7283 participants available for analysis. After excluding 20 indirect interviews in 2015, the final analytic sample comprised 7263 participants ≥60 years with complete data at both baseline (2012) and follow‐up (2015).

### 2.3 Measures

#### 2.3.1 Intrinsic capacity

**FIGURE 2:**
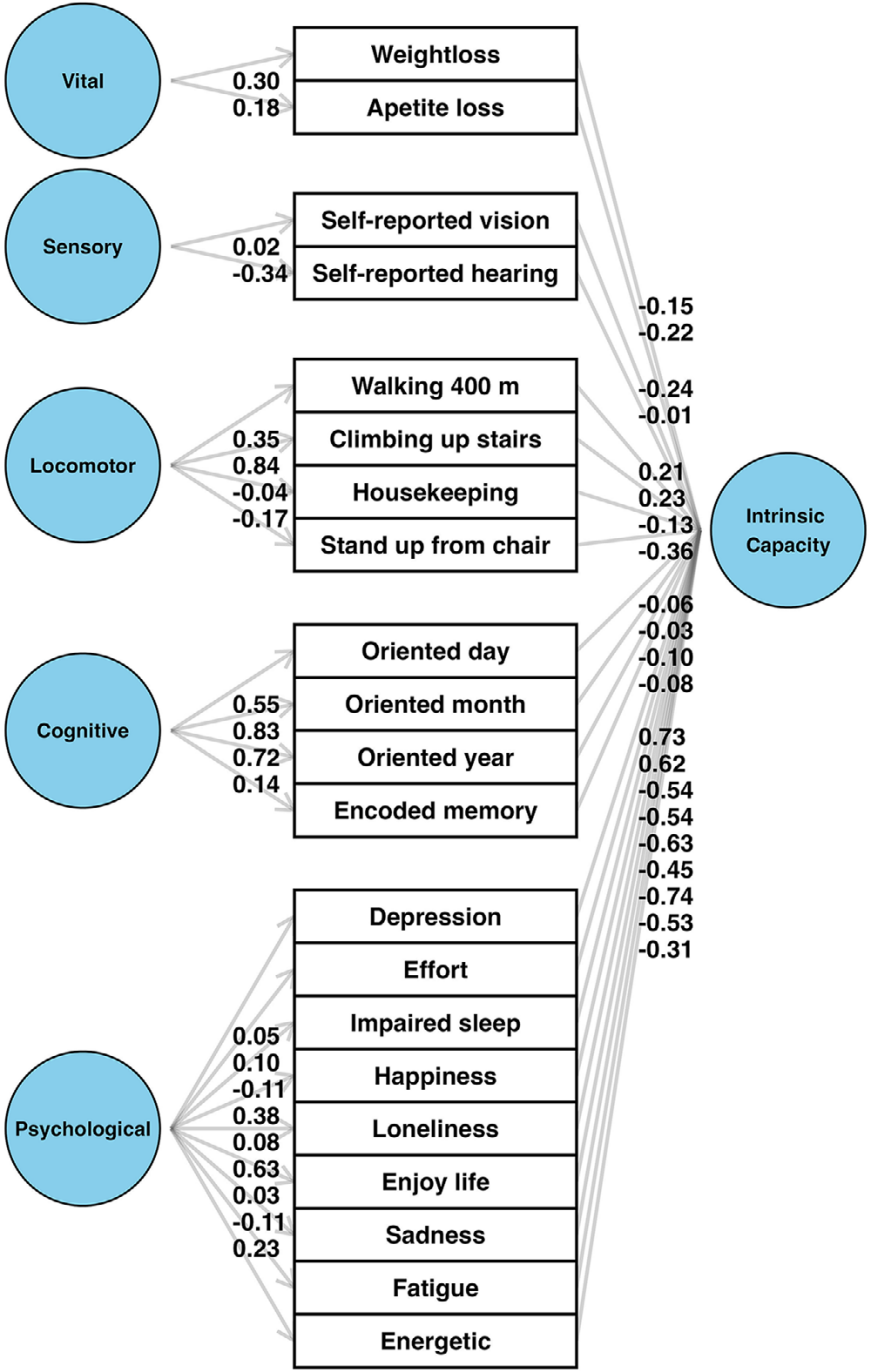
Structural equation model of intrinsic capacity domains and observed indicators. The final model included five correlated domains—vitality, sensory, locomotor, cognitive, and psychological—represented by multiple observed indicators.

A composite index of IC was constructed following the WHO ICOPE framework. An exploratory factor analysis (EFA) using scree plot inspection and oblique (promax) rotation was first performed to identify the underlying structure. Subsequently, a confirmatory factor analysis (CFA) within a structural equation modeling (SEM) framework was applied to assess model fit and domain interrelations. The final model included five correlated domains—vitality, sensory, locomotor, cognitive, and psychological—each represented by multiple observed indicators (Figure 2). Detailed standardized factor loadings and *R* ^2^ values for all indicators are presented in Supplementary Table 1. The model showed adequate goodness of fit, and standardized factor scores were generated and normalized (mean = 0, SD = 1) to produce a continuous IC variable. Higher IC scores reflected better preserved capacity, whereas lower scores indicated greater IC deficits. In addition, domain‐specific binary indicators were derived to identify the presence or absence of impairment within each domain.

Consistent with the construct‐based framework proposed by Beard et al.^10^ and with its application in prior population‐based longitudinal cohort studies.^22^, ^23^, IC was operationalized across five domains (cognitive, psychological, sensory, locomotor, and vitality) using proxy measures available in the MHAS:

1. Psychological: depressive symptoms were assessed using a modified nine‐item version of the Center for Epidemiologic Studies ‐ Depression (CES‐D) scale included in the MHAS, comprising items with dichotomous (yes/no) responses such as: *Within the past week: Respondent was depressed. experienced difficulty performing, experienced restless sleep, was happy, was lonely, enjoyed life, was sad, was tired, was energetic*. Consistent with a clinical validation study, a binary variable was created, classifying participants as having depressive symptoms if the summed score was ≥5.^24^
2. Sensorial impairment: This variable was derived from self‐reported vision (*Respondent's vision with glasses*) and hearing (*Respondent uses hearing/auditory device*). Vision impairment was coded as 1 if the participant reported impaired vision (and 0 otherwise. Hearing impairment was coded as 1 if the participant reported impaired hearing and 0 otherwise.
3. Vitality impairment: In line with prior work such as that of Gutiérrez et al.,.^18^ we defined this domain using weight (*Compared to 2 years ago: Respondent's change in weight*) and appetite loss (*Past 2 years: Respondent's loss of appetite*), which correspond to the two screening questions proposed in the WHO ICOPE guidelines.^8^ Participants were coded as impaired (1) if either or both conditions were present, and 0 otherwise.
4. Cognitive impairment: assessed with ICOPE's variables orientation (day, month, year) and verbal recall. The latter variable was assessed as impaired if participants did not recall at least three words in the immediate eight word recall test. Orientation and memory items were combined into a binary indicator; impairment (1) was assigned if deficits were present in either domain.
5. Locomotor impairment: defined from four self‐reported functional limitations. Walking (Because of health problem, difficulty walking blocks), climbing stairs (Because of health problem, difficulty with flights of stairs), housekeeping (Does respondent perform home maintenance/repairs), and rising from a chair (Because of health problem, difficulty getting up from chair). Impairment (1) was coded if limitations were present in at least one domain.

#### 2.3.2 Dementia

For self‐respondents, cognitive function in the MHAS is evaluated using an adapted version of the Cross‐Cultural Cognitive Examination (CCCE). This instrument, previously validated, assesses performance across eight domains—verbal learning, delayed recall, attention, constructional praxis, visual memory, verbal fluency, orientation, and processing speed.^25^ Dementia was defined by CCCE criteria (≤−1.5 SD in two or more domains) on reference norms, plus ≥1 limitation in instrumental activities of daily living (IADL).^26^ The latter included the ability to prepare a meal, go shopping, manage money, or take medications.

#### 2.3.3 Other covariates

Sociodemographic variables: age (continuous, years at baseline), sex (male/female), marital status (married/in union vs. single, divorced, widowed, separated), and education (categorized as no formal education, primary, or secondary/higher) were included. Since the MHAS collected participants’ sex as a binary variable (male/female) based on self‐report, no information regarding gender identity or non‐binary categories was available in the dataset. Therefore, in this study, sex refers to the participant's biological category as reported in MHAS.

Health conditions: Chronic conditions were self‐reported and coded as binary indicators: hypertension, diabetes, and heart attack. A variable of multimorbidity was constructed to indicate presence of one or more chronic conditions.

Lifestyle behaviors: Smoking status: current smoker (1) versus non‐smoker (0). Alcohol consumption: coded as 1 for current alcohol use and 0 for moderate or no use.

### 2.4 Statistical analysis

All analyses accounted for survey design (strata, clusters, and sampling weights) to ensure national representativeness.

EFA and SEM were applied to estimate IC and its five domains. CFA was then conducted to evaluate model fit and determine the final solution. Standardized IC scores (mean = 0, SD = 1) were derived and analyzed both as continuous variables and as categorical variables (tertiles).

Continuous variables were summarized using means and standard deviations, while categorical variables were described with weighted frequencies and percentages, accounting for the complex survey design (strata, clusters, and sampling weights from MHAS). For Table .1, baseline characteristics were compared across IC tertiles using weighted analysis of variance (ANOVA) for continuous variables and design‐adjusted *χ* ^2^ tests for categorical variables. Linear trends across tertiles were tested by modeling tertiles as an ordinal variable. For Table .2, changes between 2012 and 2015 were assessed with weighted paired Student's *t*‐tests for continuous variables and weighted McNemar tests for dichotomous variables. When assumptions were violated, non‐parametric alternatives (Kruskal–Wallis or Wilcoxon tests) were applied.

**TABLE 1:** Baseline characteristics of participants according to tertiles of IC score.

Variable	T1 (*n* = 2421)	T2 (*n* = 2421)	T3 (*n* = 2421)	*p*‐Value
**Sociodemographic**				
Age, years	69.23 ± 6.85	68.70 ± 6.51	67.89 ± 6.19	0.000
Sex, female %	1536 (67.71)	1192 (52.51)	1033 (45.52)	0.000
Marital status (single/divorced/widowed/separated)	903 (44.44)	664 (33.93)	545 (28.43)	0.000
Education (0–6 years)	2140 (94.19)	2142 (94.32)	2157 (94.85)	0.591
**Health conditions**				
Hypertension, %	1308 (39.78)	1063 (32.33)	917 (27.89)	0.000
Diabetes, %	637 (28.09)	507 (22.35)	448 (19.78)	0.000
Heart attack, %	112 (4.94)	84 (3.70)	66 (2.91)	0.002
Multimorbidity, %	1504 (66.34)	1268 (56.03)	1121 (49.47)	0.000
IADL dependence, %	415 (18.28)	174 (7.67)	84 (3.70)	0.000
**Lifestyle behaviors**				
Current smoking, %	757 (33.32)	896 (39.47)	906 (39.91)	0.000
Current alcohol consumption, %	397 (17.47)	530 (23.35)	617 (27.18)	0.000
**IC score**	−1.26 ± 0.50	0.28 ± 0.35	1.09 ± 0.14	0.000
**Domains of IC**				
Sensory impairment, %	785 (30.35)	1253 (45.98)	1587 (58.73)	0.000
Visual impairment, %	1258 (56.51)	1009 (45.55)	609 (27.37)	0.000
Hearing impairment, %	28 (1.23)	30 (1.32)	21 (0.92)	0.424
Locomotor impairment, %	267 (89.90)	161 (87.50)	67 (81.71)	0.128
Cognitive impairment (ICOPE), %	971 (43.66)	850 (38.08)	691 (30.99)	0.000
Vitality impairment, %	972 (43.14)	669 (29.65)	528 (23.46)	0.000
Psychological impairment, %	1765 (77.28)	1165 (45.24)	12 (0.49)	0.000

*Note*: Baseline characteristics were compared across IC tertiles using weighted ANOVA for continuous variables and design‐adjusted *χ* ^2^ tests for categorical variables.

Abbreviations: ANOVA, analysis of variance; IADL, instrumental activities daily living; IC, intrinsic capacity; ICOPE, Integrated Care for Older People; T, tertile.

**TABLE 2:** Changes in health, lifestyle, and intrinsic capacity domain variables over the 3‐year follow‐up in the total population.

Variable	Baseline	Follow‐up	*p‐*Value	∆
**Health conditions**				
Hypertension, %	3510 (48.48)	3901 (53.80)	0.027	11.14
Diabetes, %	1701 (23.49)	1884 (26.02)	0.000	10.76
Heart attack, %	269 (3.71)	321 (4.43)	0.004	19.33
Multimorbidity, %	4156 (57.37)	4507 (61.22)	0.000	8.45
IADL dependence,%	673 (9.88)	1377 (20.22)	0.000	10.34
**Lifestyle behaviors**				
Current smoking, %	2704 (37.24)	2966 (40.90)	0.000	9.73
Current alcohol consumption, %	1617 (22.27)	418 (25.85)	0.000	74.15
Intrinsic capacity score	−0.30 ± 1.03	−0.53 ± 0.83	0.000	0.232
**Domains of IC**				
Sensory impairment, %	2968 (44.05)	3381 (50.18)	0.000	13.92
Locomotor impairment, %	482 (88.12)	533 (97.44)	0.005	10.58
Cognitive impairment, (by ICOPE) %	3123 (45.85)	3588 (52.67)	0.000	14.89
Vitality impairment, %	2273 (32.56)	2449 (35.08)	0.000	7.74
Psychological impairment, %	2273 (39.77)	2449 (49.79)	0.000	25.19

*Note*: ∆ represents the relative change expressed as a percentage. Changes between 2012 and 2015 were assessed with weighted paired Student's *t*‐tests for continuous variables and weighted McNemar tests for dichotomous variables. When assumptions were violated, non‐parametric alternatives (Kruskal–Wallis or Wilcoxon tests) were applied.

Abbreviations: IADL, instrumental activities daily living; IC, intrinsic capacity; ICOPE, Integrated Care for Older People.

**FIGURE 3:**
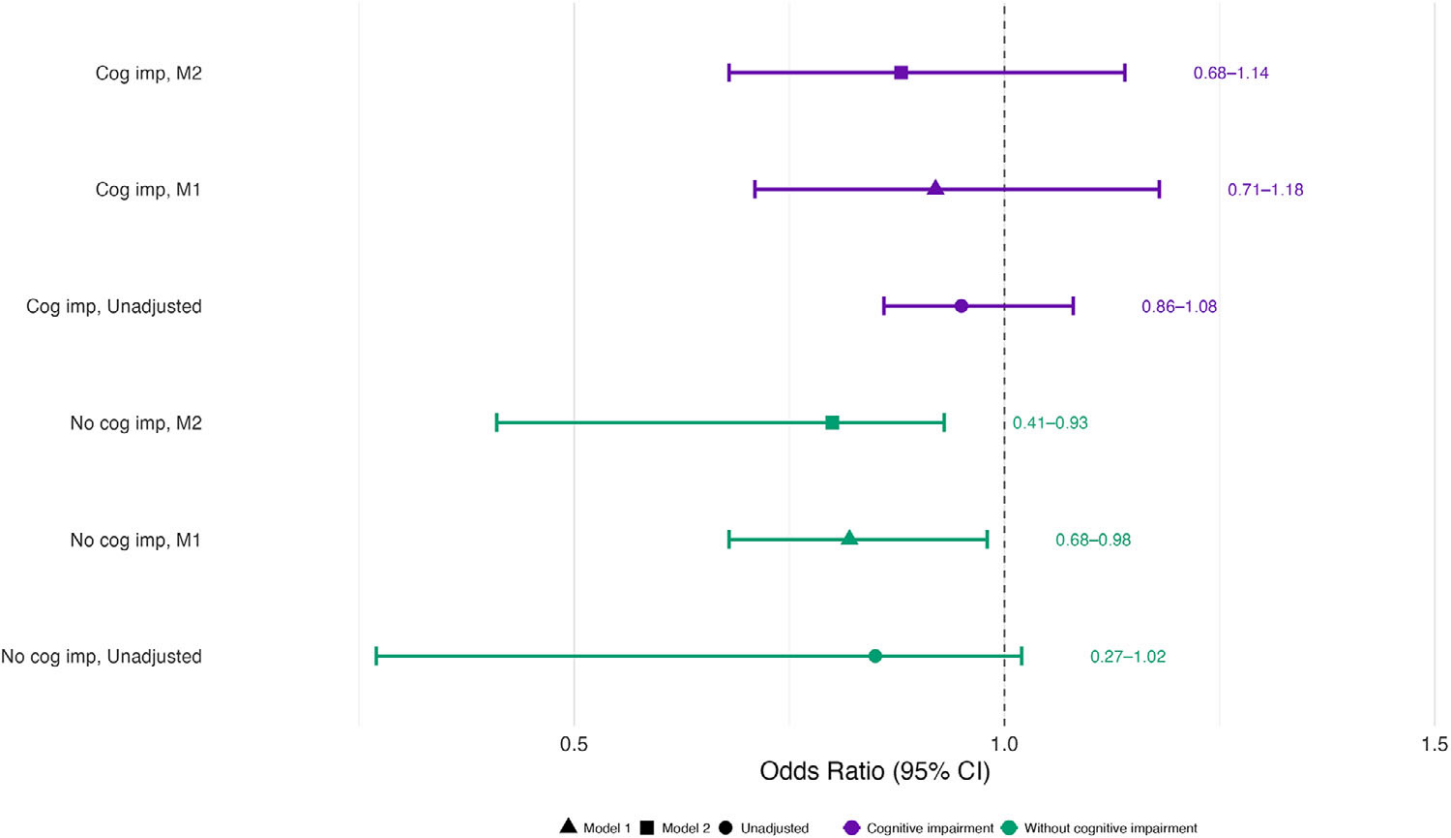
Association between intrinsic capacity and incident dementia by baseline cognitive status. Model 1 adjusted for age and sex; Model 2 additionally adjusted for marital status, education, multimorbidity, smoking, and alcohol use. CI, confidence interval; Cog imp, cognitive impairment; M1, Model 1; M2, Model 2; No cog imp, without cognitive impairment.

For Figure 3, the association between IC score and dementia incidence was evaluated using multivariable logistic regression models, adjusted for the complex survey design (svy command in Stata). Odds ratios (ORs) and 95% confidence intervals (CIs) were calculated for crude and adjusted models, including sociodemographic and health covariates. Model diagnostics included assessment of multicollinearity, residuals, linearity of continuous predictors in the logit, and overall survey‐weighted goodness‐of‐fit. We also evaluated a prespecified interaction between IC and baseline cognitive impairment, and cognitive impairment was identified as an effect modifier; therefore, all logistic models were stratified by baseline cognitive status.

**FIGURE 4:**
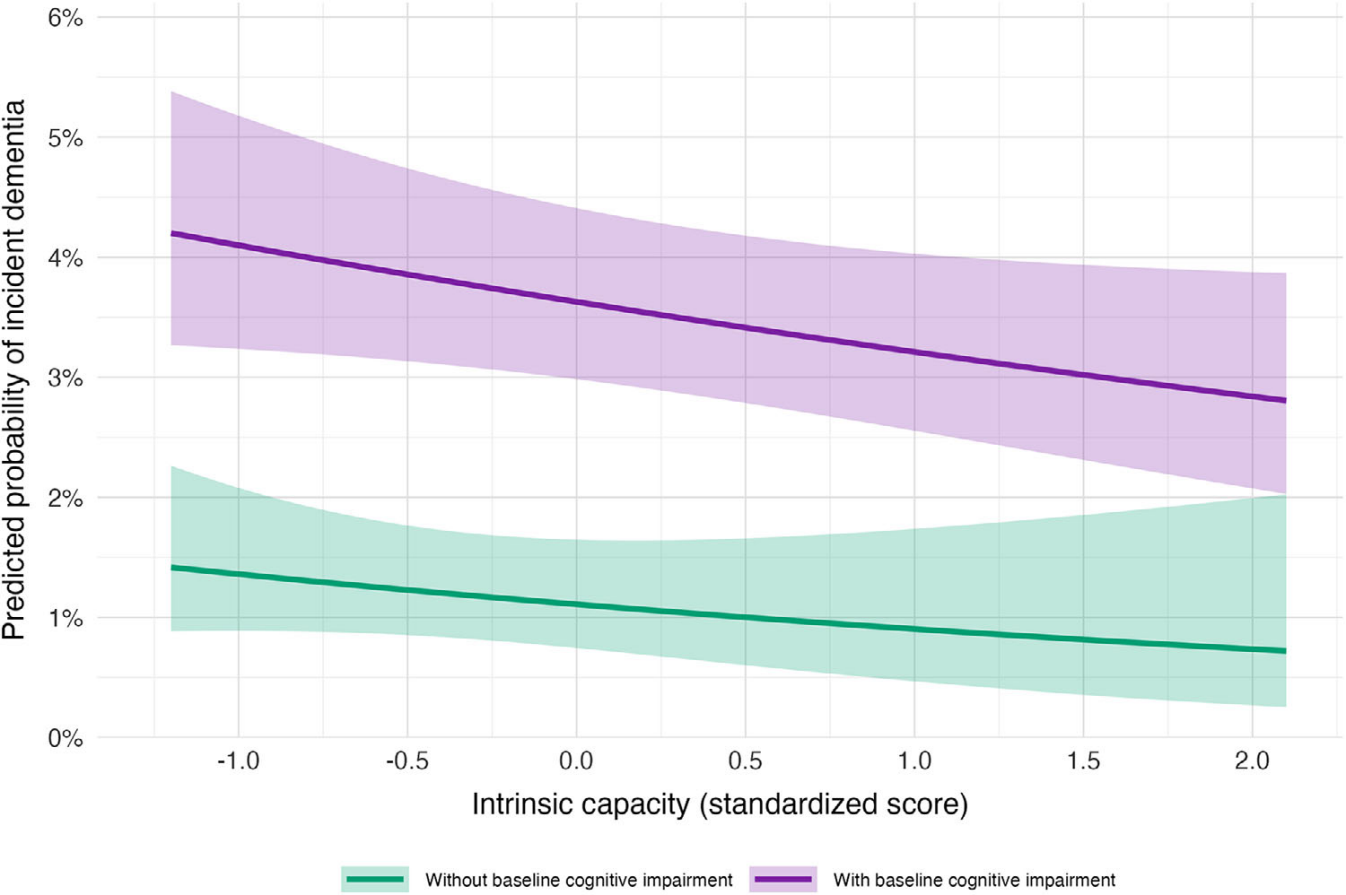
Predicted probability of incident dementia according to intrinsic capacity score, stratified by baseline cognitive status. Lines represent predicted probabilities and shaded areas indicate 95% confidence intervals.

For Figure 4, model‐based predicted probabilities of incident dementia were estimated from survey‐weighted logistic regression models, with IC score modeled as a continuous standardized variable and stratified by baseline cognitive status. The figure displays model‐based predicted probabilities and corresponding 95% CIs. Comparisons were performed using design‐adjusted *χ* ^2^ tests. Linear trend tests were also conducted by modeling IC level as an ordinal variable.

For Table S1, a CFA was conducted to define IC domains according to the WHO ICOPE framework. Standardized factor loadings and *R* ^2^ values were estimated and used to construct domain‐specific and composite IC scores, accounting for the complex survey design.

For Table S2, cross‐tabulations of incident dementia cases identified during the 3‐year follow‐up by IC level (overall and by domains) were performed. Comparisons were conducted using design‐adjusted *χ* ^2^ tests to account for the complex survey design, and linear trend tests were performed by modeling IC level as an ordinal variable.

Sensitivity analyses were conducted by recalculating the IC score excluding the cognitive domain and re‐estimating the primary models overall and stratified by baseline cognitive status (Table S3). Baseline characteristics were compared between participants retained in the analytical sample and those who died or were lost to follow‐up (Table S4). Competing‐risk regression models (Fine and Gray) were used to account for death as a competing event for incident dementia (Table S5).

Table S6 presents sensitivity analyses examining the associations between individual IC domains and incident dementia using survey‐weighted logistic regression models (crude, age‐ and sex‐adjusted, and fully adjusted).

To enable comparison with studies reporting incidence rates per person‐years, the cumulative incidence of dementia over the 3‐year follow‐up was converted into an annual incidence rate (cases per 1000 person‐years) assuming a constant hazard over time.^27^ The conversion was based on the standard relationship between cumulative risk (*p*) and incidence rate (*r*): *p* = 1 − exp(−*r* × *t*), where *t* is the follow‐up duration in years and *exp* denotes the exponential function. Solving for *r* yields: *r* = −ln(1 − *p*) / *t.  *Incidence measures were calculated independently of regression modeling and are reported in the main text.

All analyses were conducted using Stata (StataCorp, College Station, TX, USA); tests were two‐tailed, and *p*‐values < 0.05 were considered statistically significant.

## 3 RESULTS

A total of 7263 participants aged 60 years and older were included in the analytical sample; mean age was 68.92 ± 6.8 years and 55.4% were women. During the 3‐year follow‐up, 283 incident cases of dementia were identified, corresponding to a cumulative incidence of 4.15% (95% CI: 3.70–4.65) in the analytic cohort, equivalent to an incidence rate of approximately 14.2 new cases per 1000 person‐years.

The structural equation model identified five correlated domains (Figure 2). The vitality domain showed moderate loadings for weight loss (0.30) and appetite loss (0.18). The sensory domain presented low‐to‐moderate loadings for self‐reported vision (0.02) and hearing (−0.34). The locomotor domain showed the strongest contributions from climbing stairs (0.84) and walking (0.35), followed by housekeeping (−0.04) and standing up from a chair (−0.17). The cognitive domain had high loadings for orientation to month (0.83), orientation to year (0.72), and day (0.55), while memory encoding had a smaller contribution (0.14). The psychological domain showed heterogeneous loadings, with higher values for loneliness (0.63), impaired sleep (0.38), and happiness (−0.11), and lower values for other emotional indicators. All domains were positively correlated with the latent IC construct, with standardized regression weights ranging between −0.74 and 0.73 (Figure 2).

At baseline, participants in the lowest IC tertile had a higher mean age compared with those in the highest tertile. They also included a higher proportion of women, showed lower educational attainment, and had a greater prevalence of comorbidities such as hypertension and diabetes (Table .1). In addition, declines across IC domains were more frequent in the lowest tertile, particularly in the locomotor and psychological domain.

Over the 3‐year follow‐up, a marked decline was observed in IC. The mean score decreased significantly from −0.30 ± 1.03 at baseline to −0.53 ± 0.83 in 2015 (*p *< 0.001). The prevalence of impairment increased across all domains, particularly sensory (44.1% to 50.2%), cognitive (45.9% to 52.7%), and psychological (39.8% to 49.8%)  *p *< 0.001. Smaller yet significant increases were also observed for locomotor impairment (8.1% to 9.7%) and vitality impairment (32.6% to 35.1%). Health conditions worsened, with higher prevalence of hypertension (48.5% to 53.8%), diabetes (23.5% to 26.0%), heart attack (3.7% to 4.4%), and multimorbidity (57.4% to 61.2%). Lifestyle behaviors also shifted, with smoking (37.2% to 40.9%) and alcohol consumption (22.3% to 25.9%) becoming more common over time (Table .2).

In multivariable models (Figure 3), higher baseline IC scores were inversely associated with incident dementia. When stratified by baseline cognitive status, no statistically significant associations were observed among participants with baseline cognitive impairment (Model 1: OR = 0.92, 95% CI: 0.71–1.18; *p* = 0.527; Model 2: OR = 0.88, 95% CI: 0.68–1.14; *p* = 0.366). In contrast, among participants without baseline cognitive impairment, higher IC scores were associated with a lower risk of incident dementia, with stronger and statistically significant associations after adjustment for sociodemographic and health‐related variables (Model 1: OR = 0.89, 95% CI: 0 0.80–0.99; *p* = 0.036; Model 2: OR = 0.62, 95% CI: 0.41–0.93; *p* = 0.030).

Figure 4 presents the adjusted probability of incident dementia according to standardized IC scores, stratified by baseline cognitive status. Among participants without baseline cognitive impairment, lower IC scores were associated with higher predicted probabilities of dementia. Participants with baseline cognitive impairment showed higher predicted probabilities of incident dementia across the IC spectrum. Predicted probabilities were lower at higher IC scores in this subgroup, although differences were not statistically significant.

Descriptive analyses (Table 2) showed that participants who developed dementia had a higher proportion of low or medium IC compared with those who did not develop dementia (67.1% vs. 32.9%; *p* = 0.028). Across domains, cognitive impairment was more frequent among participants who developed dementia (50.4% vs. 37.0%; *p *< 0.001), while differences for sensory and psychological impairments were of borderline statistical significance (*p* = 0.059), and no significant differences were observed for vitality impairment.

In the population free of dementia at baseline, higher non‐cognitive IC remained significantly associated with a lower risk of incident dementia across crude and fully adjusted models (adjusted OR = 0.71, 95% CI 0.57–0.87; Table S3). When analyses were stratified by baseline cognitive status, associations between non‐cognitive IC and incident dementia differed across strata: Among cognitively unimpaired participants, associations were not statistically significant, whereas among participants with baseline cognitive impairment, higher non‐cognitive IC was inversely associated with incident dementia (fully adjusted OR = 0.63, 95% CI 0.50–0.79).

Baseline comparisons between participants retained in the analytical sample and those who died or were lost to follow‐up are presented in Table S4. Participants who died or were lost to follow‐up were older, more frequently women, and had lower educational attainment compared with those retained in the analytical sample. They also showed a higher prevalence of IADL dependence and poorer baseline status across cognitive, vitality, and psychological domains. Differences in overall IC score distribution between groups were limited, whereas participants who died or were lost to follow‐up had higher frequencies of impairment across multiple IC domains at baseline. Competing‐risk regression analyses accounting for death as a competing event are shown in Table S5.

Multivariable regression models were conducted to estimate adjusted associations between IC domains and incident dementia. In multivariable models (Table S6), higher locomotor IC was independently associated with lower odds of incident dementia after adjustment for age and sex (OR = 0.80; 95% CI: 0.66–0.96), and this association remained significant in the fully adjusted model (OR = 0.79; 95% CI: 0.66–0.95). In contrast, no significant associations were observed for the sensory, psychological, or cognitive domains.

## 4 DISCUSSION

Within the WHO framework for healthy aging, IC represents the physiological and cognitive reserves that sustain autonomy across the lifespan. Our findings extend this concept to dementia risk: over a 3‐year follow‐up, participants with higher IC scores were significantly less likely to develop dementia, whereas those in the lowest IC tertile were older, less educated, and exhibited more comorbidities. These results underscore the relevance of IC as a holistic, clinically meaningful construct that captures early vulnerability to neurodegenerative processes beyond traditional factors.

The IC index constructed in this study followed the WHO ICOPE framework and demonstrated an adequate factorial structure encompassing five correlated domains: vitality, sensory, locomotor, cognitive, and psychological. This multidimensional model aligns with prior studies validating IC across different populations.^7^, ^9^, ^10^, ^12^ By combining exploratory and confirmatory factor analyses, we ensured construct validity and internal coherence, consistent with approaches used in longitudinal cohorts such as the UK Biobank.^6^ and multicountry ICOPE‐based studies.^10^, ^19^, ^28^, ^29^ The derivation of a standardized continuous score (mean = 0, SD = 1) further enhances its utility for quantitative analyses and longitudinal tracking, facilitating examination of associations with adverse outcomes such as dementia.

The estimated dementia incidence rate of approximately 14.2 cases per 1000 person‐years was within the lower range reported in other Latin American cohorts. The 10/66 Dementia Research Group reported higher incidence rates across multiple sites.^30^, whereas the Maracaibo Aging Study observed lower rates, particularly in younger cohorts.^31^ Differences across studies likely reflect variation in diagnostic criteria, assessment methods, age structure, and follow‐up duration, with algorithm‐based definitions generally yielding lower incidence estimates.

Consistent with prior literature, individuals with lower IC tended to be older, less educated, predominantly women, and to present greater multimorbidity.^10^, ^19^, ^32^ Locomotor limitations were the most prevalent impairment in the lowest IC tertile, followed by psychological and cognitive domains, mirroring findings from multicohort and ICOPE‐based studies.^28^, ^29^ In middle‐income settings such as Mexico, these patterns may reflect cumulative physical strain, limited access to rehabilitative care, and chronic psychosocial stressors across the life course.^32^, ^33^, ^34^, ^35^

The association between IC and incident dementia was most evident among individuals without baseline cognitive impairment. Although associations were directionally similar among those with baseline impairment, estimates did not reach statistical significance and should be interpreted cautiously. Given the relatively short follow‐up period, some participants who developed dementia may have already been in a prodromal stage at baseline, particularly those with pre‐existing cognitive impairment. This could partially explain the weaker and less precise associations observed in this subgroup, as the neurodegenerative process may have already been underway. These findings suggest that the predictive value of IC appears strongest in earlier stages of aging, before cognitive impairment becomes established.

These findings are consistent with previous research.^36^, ^37^ showing that lower IC at baseline is associated with poorer cognitive trajectories and functional outcomes in older adults. Evidence from the Multidomain Alzheimer Preventive Trial supports IC as an early marker of cognitive vulnerability, linking lower baseline IC to worse cognitive performance and higher levels of neurodegeneration biomarkers.^36^ Other studies suggest that non‐cognitive IC domains are more strongly related to functional outcomes in earlier stages of cognitive decline, with weaker associations as neurodegeneration progresses.^37^

In the present analysis, sensory and psychological domain impairments showed trends toward association with dementia development. Sensory deficits, particularly hearing and vision loss, have been consistently linked to dementia risk through mechanisms involving social isolation, increased cognitive load, reduced environmental stimulation, and depletion of cognitive reserve.^38^, ^39^, ^40^, ^41^ Psychological health may further contribute to cognitive reserve by promoting healthier behaviors, reducing chronic stress, and mitigating inflammation.^42^

It must be noted that, in descriptive analyses based on design‐adjusted *χ* ^2^ tests, participants who developed dementia during follow‐up showed a higher prevalence of cognitive impairment and a lower proportion of high IC scores at baseline, whereas differences in vitality, locomotor, sensory, and psychological impairments were small and not consistently statistically significant. In contrast, multivariable survey‐weighted models accounting for sociodemographic and health covariates (Table S6) demonstrated that locomotor IC was the only domain independently associated with incident dementia, while associations for sensory, vitality, psychological, and cognitive domains were attenuated after adjustment. These findings are consistent with previous evidence linking locomotor decline to cognitive deterioration and dementia risk.^43^, ^44^, ^45^, and further supported by studies on Motoric Cognitive Risk (MCR) syndrome, which integrates slow gait and cognitive complaints as a preclinical marker of dementia and has been associated with increased risk of cognitive impairment and dementia across diverse populations, including Mexican older adults.^43^ This body of evidence supports the biological plausibility that locomotor function may capture early neurodegenerative and vascular changes affecting shared frontal‐subcortical networks involved in both gait and cognition. These findings highlight the importance of distinguishing baseline vulnerability patterns from independent domain‐specific effects identified in adjusted longitudinal analyses.

In addition, relatively low or heterogeneous indicator loadings observed for some domains may have reduced sensitivity to detect domain‐specific longitudinal associations. This has implications for the construct validity of domain‐specific IC measures and suggests that caution is needed when interpreting domain‐level associations. Future studies should incorporate more refined and standardized indicators to improve the sensitivity and comparability of IC domains across populations.

This study has several strengths, including its longitudinal design, stratification by baseline cognitive status, and adjustment for multiple sociodemographic and health covariates. Importantly, this is the first nationally representative analysis in Mexico to demonstrate an association between IC and incident dementia, providing novel evidence from a middle‐income country where longitudinal dementia research remains limited. These findings support the integration of IC into healthy aging programs to improve early dementia risk identification and guide multidimensional care, particularly in resource‐limited settings.

Several limitations should also be acknowledged. Variability in IC domain measurement and reliance on an algorithmic definition of dementia—without clinical adjudication or confirmation using neurodegenerative biomarkers—may have led to under‐ascertainment and potential non‐random misclassification, particularly influenced by education or sensory impairments. Sensitivity analyses were conducted, including exclusion of prevalent dementia, stratification by baseline cognitive status, and recalculation of IC excluding the cognitive domain. Nevertheless, residual reverse causation cannot be fully excluded, as lower IC scores at baseline may reflect underlying prodromal neurodegenerative processes—affecting cognitive, psychological, and functional domains—prior to clinical dementia diagnosis. Moreover, heterogeneity in IC operationalization and indicator selection remains a challenge for broader application.^46^ Grip strength, a key objective indicator of the vitality domain, was only available in a small subsample and, therefore, could not be included in this analysis, which may have limited the sensitivity of this domain.

Concerns regarding circularity arise because cognition is included within IC while dementia is defined using cognitive criteria. However, prior longitudinal evidence indicates that IC retains predictive validity even when cognition is excluded.^36^ Consistent with this evidence, our sensitivity analyses showed that non‐cognitive IC remained inversely associated with incident dementia overall. Differences between the main analysis and the sensitivity analysis excluding the cognitive domain may reflect stage‐dependent effects of IC. In the primary analysis, inclusion of the cognitive domain likely improves detection of early vulnerability among individuals without baseline cognitive impairment, when subtle changes may still be emerging.

In contrast, excluding the cognitive domain may allow non‐cognitive domains to better capture progression among individuals with established impairment, where functional decline is more pronounced. These findings suggest complementary roles of cognitive and non‐cognitive domains across stages of cognitive decline, rather than true inconsistency between analyses. Moreover, although IC is conceptualized as a multidimensional construct, its association with incident dementia may be partly driven by the cognitive domain. Nevertheless, the inclusion of multiple physical and mental domains supports the value of IC as an integrated marker of overall functional reserve and vulnerability, rather than a purely cognitive measure.

Attrition and death represent inherent challenges in aging cohorts. Participants who died or were lost to follow‐up were older and had worse baseline profiles, and exclusion of these individuals may have introduced survival bias, as death is a competing risk for dementia. This may have led to underestimation of observed associations. Nevertheless, the consistency of findings across adjusted models and sensitivity analyses supports their robustness, and future studies using competing risk approaches are warranted.^47^

Overall, our findings support IC as a multidimensional marker associated with incident dementia, particularly in cognitively unimpaired older adults. The integration of multiple functional domains into a single IC construct reflects the cumulative burden of age‐related declines and their interaction, which may not be fully captured by domain‐specific measures alone. As such, IC provides a pragmatic and conceptually coherent framework for early risk stratification, multidimensional care planning, and prevention strategies in the context of dementia risk. Future studies should confirm these findings across diverse populations, explore underlying mechanisms, and evaluate interventions designed to maintain or improve IC as a strategy to promote healthy aging. Importantly, longitudinal analyses using time‐to‐event models with competing risk approaches will be essential to more fully account for mortality when estimating dementia risk, while also addressing ongoing challenges in IC operationalization.^46^ and competing risks such as death.^47^

## CONFLICT OF INTEREST STATEMENT

The authors declare no conflicts of interest. Author disclosures are available in the Supporting Information.

## CONSENT STATEMENT

This study used a publicly available dataset from the MHAS (www.mhas.org), for which informed consent had been previously obtained by the original investigators. No additional consent was required for the present analysis.

## Supporting information




**Supporting Information**: alz71578‐sup‐0001‐ICMJE.pdf

**Supporting Information**: alz71578‐sup‐0002‐TableS1.docx

**Supporting Information**: alz71578‐sup‐0003‐TableS2.docx

**Supporting Information**: alz71578‐sup‐0004‐TableS3.docx

**Supporting Information**: alz71578‐sup‐0005‐TableS4.docx

**Supporting Information**: alz71578‐sup‐0006‐TableS5.docx

**Supporting Information**: alz71578‐sup‐0007‐TableS6.docx
