# Supplementary material for: Association between intrinsic capacity and dementia risk in older Mexicans
Source: Alzheimers Dement. 2026 Jun 17;22(6):e71578. doi: 10.1002/alz.71578 (PMC13275326; doi:10.1002/alz.71578)
Supplement: Supplementary file 2 — Supporting Information: alz71578‐sup‐0002‐TableS1.docx [file ALZ-22-e71578-s003.docx]

### **Supplementary Table 1.** Confirmatory factor analysis results for intrinsic capacity domains.

| Domain / Indicator | Standardized loading | *R²* |
| --- | --- | --- |
| Psychological domain |  |  |
| Depressed | 0.72 | 0.523 |
| Effort | 0.60 | 0.358 |
| Restless sleep | -0.52 | 0.273 |
| Happiness | -0.56 | 0.318 |
| Loneliness | 0.64 | 0.409 |
| Enjoy life | -0.48 | 0.227 |
| Sadness | 0.75 | 0.566 |
| Tired | -0.52 | 0.265 |
| Energetic | -0.33 | 0.108 |
| Cognitive domain |  |  |
| Orientation to day | 0.55 | 0.308 |
| Orientation to month | 0.83 | 0.671 |
| Orientation to year | 0.73 | 0.541 |
| Memory | 0.14 | 0.022 |
| Locomotor domain |  |  |
| Walking | 0.38 | 0.141 |
| Climbing stairs | 0.43 | 0.188 |
| Housekeeping | -0.17 | 0.028 |
| Standing up from a chair | -0.52 | 0.269 |
| Sensory domain |  |  |
| Vision | 0.60 | 0.354 |
| Hearing | 0.00 | 0.000 |
| Vitality domain |  |  |
| Weight loss | 0.24 | 0.057 |
| Appetite loss | 0.36 | 0.130 |

NOTE. Standardized factor loadings and *R²* values were estimated and used to construct domain-specific and composite IC scores.
