# Supplementary material for: Association between intrinsic capacity and dementia risk in older Mexicans
Source: Alzheimers Dement. 2026 Jun 17;22(6):e71578. doi: 10.1002/alz.71578 (PMC13275326; doi:10.1002/alz.71578)
Supplement: Supplementary file 3 — Supporting Information: alz71578‐sup‐0003‐TableS2.docx [file ALZ-22-e71578-s002.docx]

**Supplementary Table 2.** Distribution of incident dementia cases and non-cases according to intrinsic capacity level (overall and by domains).

| **Variable** | **Dementia Yes (n=283)** | **Dementia No (n=6980)** | ***p-value*** |
| --- | --- | --- | --- |
| High IC score* | 93 (32.86) | 2178 (33.35) |  |
| Low/medium IC score* | 190 (67.14) | 4353 (66.65) | .028 |
| Sensory impairment, % | 119 (42.05) | 2384 (36.53) | .059 |
| Locomotor impairment, % | 30 (85.71) | 465 (88.07) | .171 |
| Cognitive impairment (ICOPE), % | 139 (50.36) | 2373 (37.02) | .000 |
| Vitality impairment, % | 98 (32.09) | 2071 (31.97) | .327 |
| Psychological impairment, % | 119 (42.05) | 2384 (36.53) | .059 |

NOTE. Values are n (%), column percentages. Dementia cases are incident cases identified during follow-up. Design-adjusted χ² tests were used. *High, medium, and low intrinsic capacity categories were defined according to tertiles of the baseline score distribution.
Abbreviations: IC, Intrinsic capacity; ICOPE, Integrated Care for Older People.
