# Supplementary material for: Association between intrinsic capacity and dementia risk in older Mexicans
Source: Alzheimers Dement. 2026 Jun 17;22(6):e71578. doi: 10.1002/alz.71578 (PMC13275326; doi:10.1002/alz.71578)
Supplement: Supplementary file 4 — Supporting Information: alz71578‐sup‐0004‐TableS3.docx [file ALZ-22-e71578-s001.docx]

**Supplementary Table 3.** Sensitivity analysis of the association between intrinsic capacity (excluding cognition) and incident dementia, overall and stratified by baseline cognitive status.

| **Population / Model** | **OR (95% CI)** | ***p-value*** |
| --- | --- | --- |
| **Total population** | | |
| Crude | 0.73 (0.60–0.90) | 0.003 |
| Adjusted Model 1 † | 0.71 (0.58–0.88) | 0.002 |
| Adjusted Model 2 ‡ | 0.71 (0.57–0.87) | 0.001 |
| **No baseline cognitive impairment** | | |
| Crude | 1.07 (0.56–2.04) | 0.839 |
| Adjusted Model 1 † | 1.08 (0.56–2.07) | 0.813 |
| Adjusted Model 2 ‡ | 1.14 (0.58–2.25) | 0.703 |
| **Baseline cognitive impairment** |  |  |
| Crude | 0.64 (0.52–0.80) | 0.000 |
| Adjusted Model 1 † | 0.65 (0.52–0.81) | 0.000 |
| Adjusted Model 2 ‡ | 0.63 (0.50–0.79) | 0.000 |

NOTE. Model 1 adjusted for age and sex. Model 2 additionally adjusted for marital status, comorbidity, educational level, alcohol consumption, and smoking status.
Abbreviations: OR, Odds Ratio; CI, Confidence interval.
