# Supplementary material for: Association between intrinsic capacity and dementia risk in older Mexicans
Source: Alzheimers Dement. 2026 Jun 17;22(6):e71578. doi: 10.1002/alz.71578 (PMC13275326; doi:10.1002/alz.71578)
Supplement: Supplementary file 5 — Supporting Information: alz71578‐sup‐0005‐TableS4.docx [file ALZ-22-e71578-s004.docx]

**Supplementary Table 4.** Baseline characteristics by follow-up status (included vs deaths/lost).

| **Variable** | **Included  (n=7,263)** | **Deaths/Lost (n=1,141)** | ***p*-value** |
| --- | --- | --- | --- |
| **Sociodemographic** | | | |
| Age, years (mean ± SD) | 68.56 ± 6.54 | 72.35 ± 8.37 | 0.000 |
| Sex, female % | 3,595 (54.88) | 426 (61.21) | 0.001 |
| Marital status (single/divorced/widowed/separated), % | 2,070 (31.6) | 42 (15.97) | 0.001 |
| Education (0–6 years), % | 5,790 (88.38) | 651 (93.53) | 0.000 |
| **Health conditions** | | | |
| Hypertension, % | 3,149 (48.07) | 355 (51.01) | 0.336 |
| Diabetes, % | 1,523 (23.25) | 176 (25.29) | 0.250 |
| Heart attack, % | 252 (3.85) | 17 (2.44) | 0.176 |
| Multimorbidity, % | 3,730 (56.94) | 417 (59.91) | 0.312 |
| IADL dependence, % | 638 (9.74) | 141 (20.26) | 0.000 |
| **Lifestyle behaviors** | | | |
| Current smoking, % | 2,473 (37.75) | 227 (32.61) | 0.025 |
| Current alcohol consumption, % | 1,489 (22.73) | 122 (17.53) | 0.006 |
| Intrinsic capacity score* | −0.38[−0.96,0.75] | 0.18 [−0.70,1.25] | 0.000 |
| **Domains of intrinsic capacity** | | | |
| Sensory impairment, % | 2,809 (43.85) | 144(44.02) | 0.958 |
| Visual impairment, % | 2,764 (42.19) | 332 (47.70) | 0.006 |
| Hearing impairment, % | 76 (1.16) | 15 (2.16) | 0.077 |
| Locomotor impairment, % | 3,486 (53.23) | 432 (62.16) | 0.534 |
| Cognitive impairment (ICOPE), % | 4,201 (64.13) | 536 (77.01) | 0.000 |
| Vitality impairment, % | 2,074 (31.66) | 268 (38.51) | 0.000 |
| Psychological impairment, % | 2,391 (36.50) | 351 (50.43) | 0.000 |

NOTE. Values are expressed as mean ± SD, median [IQR], or n (%). Group comparisons were performed using Student’s *t* test, Wilcoxon rank-sum test, or Pearson’s chi-square test, as appropriate.
Abbreviations: SD, Standard deviation; IADL, Instrumental activities of daily living; ICOPE, Integrated Care for Older People; IQR, Interquartile range.
