# Supplementary material for: Association between intrinsic capacity and dementia risk in older Mexicans
Source: Alzheimers Dement. 2026 Jun 17;22(6):e71578. doi: 10.1002/alz.71578 (PMC13275326; doi:10.1002/alz.71578)
Supplement: Supplementary file 6 — Supporting Information: alz71578‐sup‐0006‐TableS5.docx [file ALZ-22-e71578-s007.docx]

**Supplementary Table 5**. Intrinsic capacity and risk of incident dementia after accounting for differential mortality.

| Variable | SHR | 95% CI | *p-value* |
| --- | --- | --- | --- |
| Intrinsic capacity | 0.82 | 0.74–0.92 | 0.001 |
| Age (years) | 1.07 | 1.05–1.08 | <0.001 |
| Female sex | 0.73 | 0.56–0.97 | 0.029 |
| Low education (0–6 years) | 1.55 | 0.60–3.96 | 0.364 |
| Marital status (single/divorced/widowed) | 0.84 | 0.65–1.09 | 0.191 |
| Current smoking | 1.16 | 0.89–1.51 | 0.271 |
| Current alcohol consumption | 0.94 | 0.70–1.27 | 0.696 |

NOTE. Subdistribution hazard ratios from Fine and Gray models with death as a competing event.

Abbreviations: SHR, Subdistribution hazard ratios; CI, Confidence interval.
