# Supplementary material for: Association between intrinsic capacity and dementia risk in older Mexicans
Source: Alzheimers Dement. 2026 Jun 17;22(6):e71578. doi: 10.1002/alz.71578 (PMC13275326; doi:10.1002/alz.71578)
Supplement: Supplementary file 7 — Supporting Information: alz71578‐sup‐0007‐TableS6.docx [file ALZ-22-e71578-s006.docx]

| Intrinsic Capacity Domain (z-score) | Crude OR (95% CI) | Model 1† OR (95% CI) | Model 2‡ OR (95% CI) |
| --- | --- | --- | --- |
| Vitality | 0.87 (0.72 – 1.06) | 0.97 (0.80 – 1.17) | 1.01 (0.83 – 1.23) |
| Sensory | 0.80 (0.66 – 0.98) | 0.92 (0.74 – 1.14) | 0.92 (0.74 – 1.13) |
| Locomotion | 0.78 (0.66 – 0.92) | 0.80 (0.66 – 0.96) | 0.79 (0.66 – 0.95) |
| Psychological | 0.88 (0.73 – 1.06) | 0.98 (0.80 – 1.21) | 1.00 (0.81 – 1.23) |
| Cognitive | 0.58 (0.38 – 0.89) | 0.75 (0.48 – 1.17) | - 1. (0.48 – 1.15) |

**Supplementary Table 6.** Association between Intrinsic Capacity domains and incident dementia.

NOTE**.** Model 1† adjusted for age and sex. Model 2‡ adjusted for age, sex, marital status, education, smoking, alcohol consumption, and comorbidity.
Abbreviations: CI, Confidence Interval; OR, Odds ratio.
